# Supplementary material for: Ranking of non-coding pathogenic variants and putative essential regions of the human genome
Source: Nat Commun. 2019 Nov 20;10:5241. doi: 10.1038/s41467-019-13212-3 (PMC6868241; doi:10.1038/s41467-019-13212-3)
Supplement: Supplementary file 2 — Supplementary Information [file 41467_2019_13212_MOESM2_ESM.pdf]

Supplementary materials

Ranking of non-coding pathogenic variants and putative essential regions of the human genome

Wells et al.

Figures

Supplementary Figure 1. Study design.

Supplementary Figure 2. Genomic element distribution among pathogenic and control variants.

Supplementary Figure 3. ncER performance comparison with existing metrics.

Supplementary Figure 4. ncER generalization to independent variant sets.

Supplementary Figure 5. High score ncER regions distribution in the genome.

Supplementary Figure 6. The GO terms enrichment for biological processes in high score ncER regions.

Supplementary Figure 7. GWAS catalog variants ncER distribution.

Supplementary Figure 8. Comparison of experimental CRISPRi functional assays with *in silico* predictions of deleteriousness.

Supplementary Figure 9. Comparison of experimental CREST-seq functional assays with *in silico* predictions of expression.

Supplementary Figure 10. CREST-seq peaks enrichment in high score ncER regions.

Supplementary Figure 11. Cumulative essential territory within transmitted deletions.

Supplementary Figure 12. Size distribution of *cis*-regulatory transmitted deletions.

Supplementary Figure 13. Fraction of transmitted deletions with high score ncER domains.

Supplementary Figure 14. Enrichment in high score ncER regions for mouse functional enhancers.

Supplementary Figure 15. ncER input feature signals across IHH locus.

Tables

Supplementary Table 1. Predictive performance and accuracy of ncER compared to CRISPRi functional assays.

Supplementary Table 2. Predictive performance and accuracy of ncER compared to CREST-seq functional assays.

Supplementary Figure 1. Study design.

CREST-seq= *cis*-regulatory element scan by tiling-deletion and sequencing.

CRISPRi= clustered regularly interspaced short palindromic repeats (CRISPR) interference.

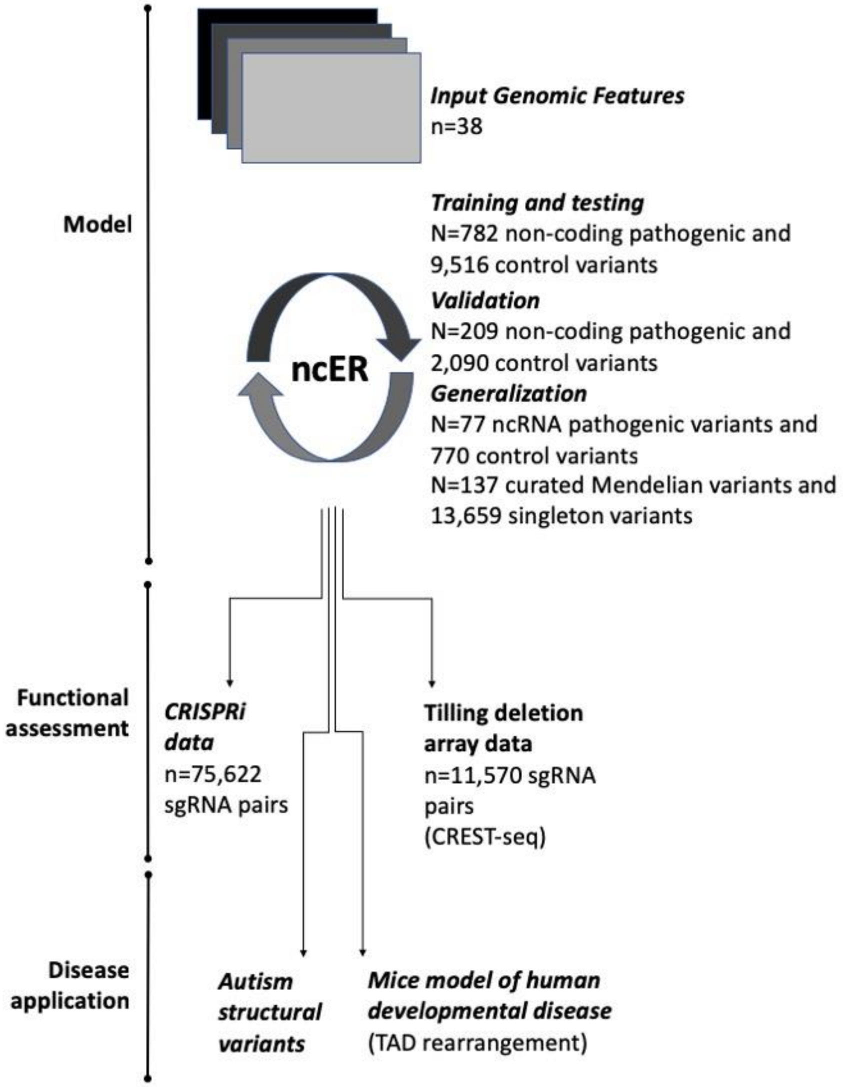

Supplementary Figure 2. Genomic element distribution among pathogenic and control variants. Bar plot displaying the cumulative fraction of genomic element represented in each set of pathogenic and matched control variants (indicated at the bottom of the bars). The total number of variants per set is displayed at the top of the bars. The genomic elements appear in the bar plot in the same order as in the legend. ncRNA, non-coding RNA. CDS, coding sequence. UTR, untranslated region.

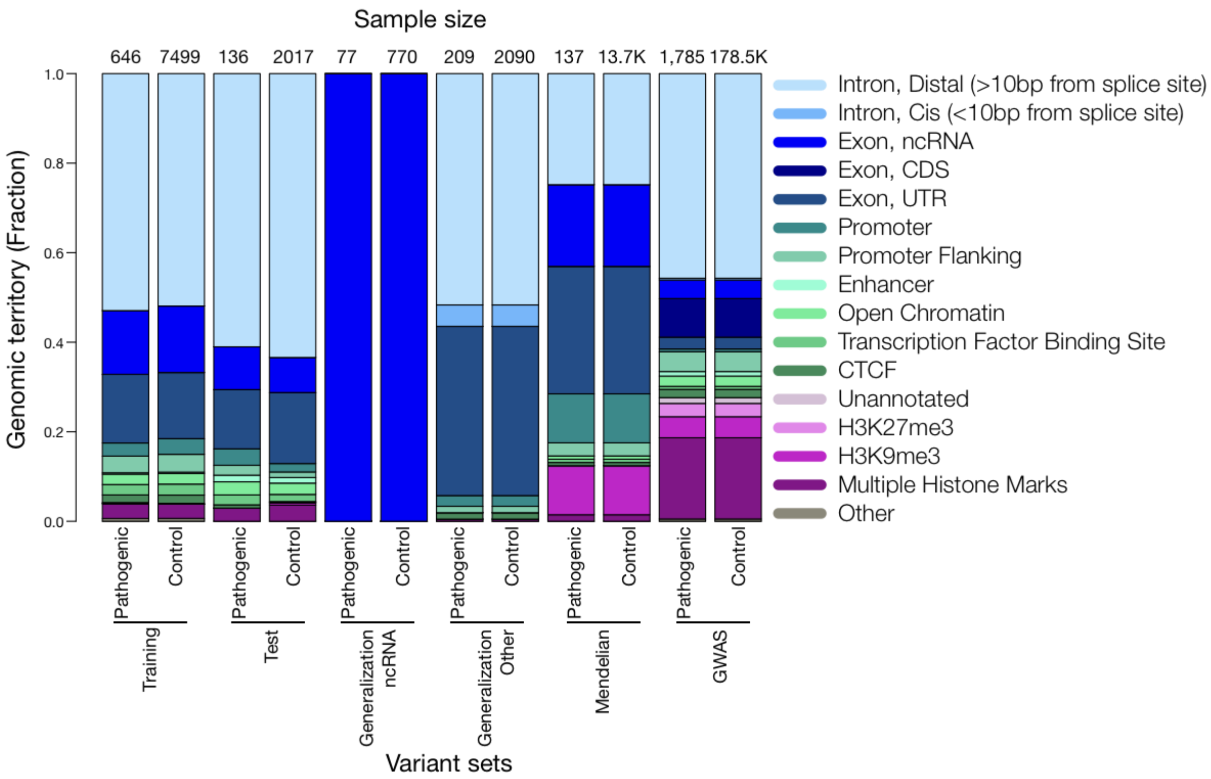

Supplementary Figure 3. ncER performance comparison with existing metrics.

Performance ROC-AUC (**panel A**) and PR-AUC (**panel B**) on the test set (N=136 pathogenic and N=2,017 control) for ncER and all individual existing metrics used in the model. The color codes and AUC for each metric are shown in the legend.

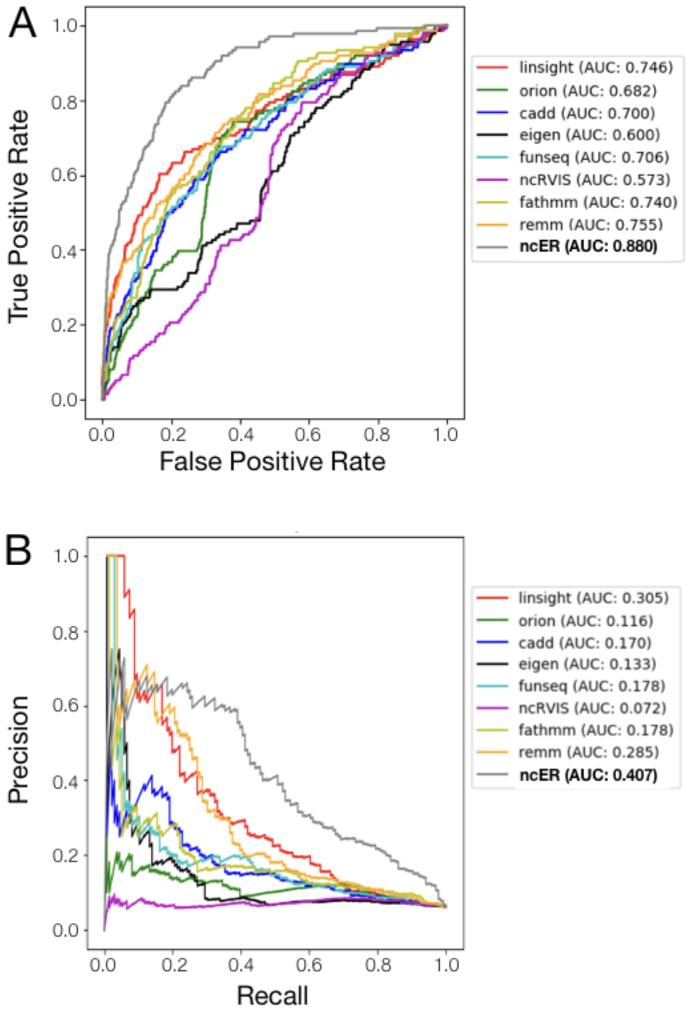

Supplementary Figure 4. ncER generalization to independent variant sets.

Performance ROC-AUC (**panel A**) and PR-AUC (**panel B**) for the independent set of left out non-coding HGMD and ClinVar variants mapping to ncRNAs (N=77 pathogenic and N=770 control). Performance ROC-AUC (**panel C**) and PR-AUC (**panel D**) for the independent set of left out non-coding HGMD and ClinVar variants mapping outside of ncRNAs (Others; N=209 pathogenic and N=2,090 control). The color codes and AUC for each metric are shown in the legend. ncRNA, non-coding RNA.

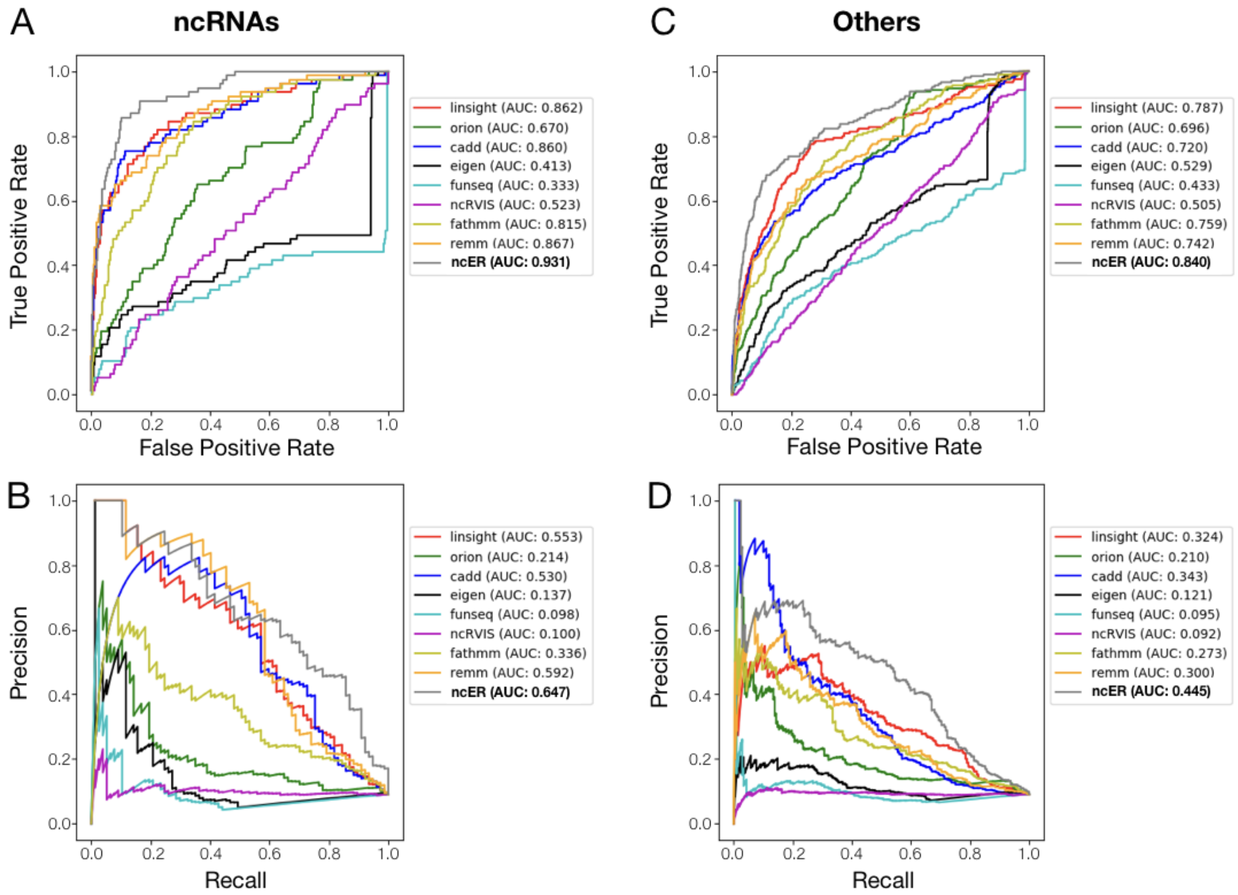

Supplementary Figure 5. Distribution of high score ncER regions in the genome.

Panel A. Bar plot displaying the cumulative territory covered by each non-coding element family at different percentile threshold (indicated at the bottom of the bars). The total non-coding genomic territory is displayed at the top of the bars. The percentiles are based on the rank of ncER values. The elements appear in the bar plot in the same order as in the legend. **Panel B**. Bar plot displaying the cumulative territory covered at each percentile threshold per genomic element (indicated at the bottom of the bars). A grey dotted line placed at 0.05 indicates the expected fraction of territory at ncER percentiles >=95 in the absence of enrichment or depletion. Genomic elements are ordered according to their relative enrichment in high score ncER regions. **Panel C**. Size distribution of high score ncER regions defined at different ncER percentile thresholds (indicated by the color code in the top-right of the figure). GW, genome-wide. TFBS, Transcription factor binding site.

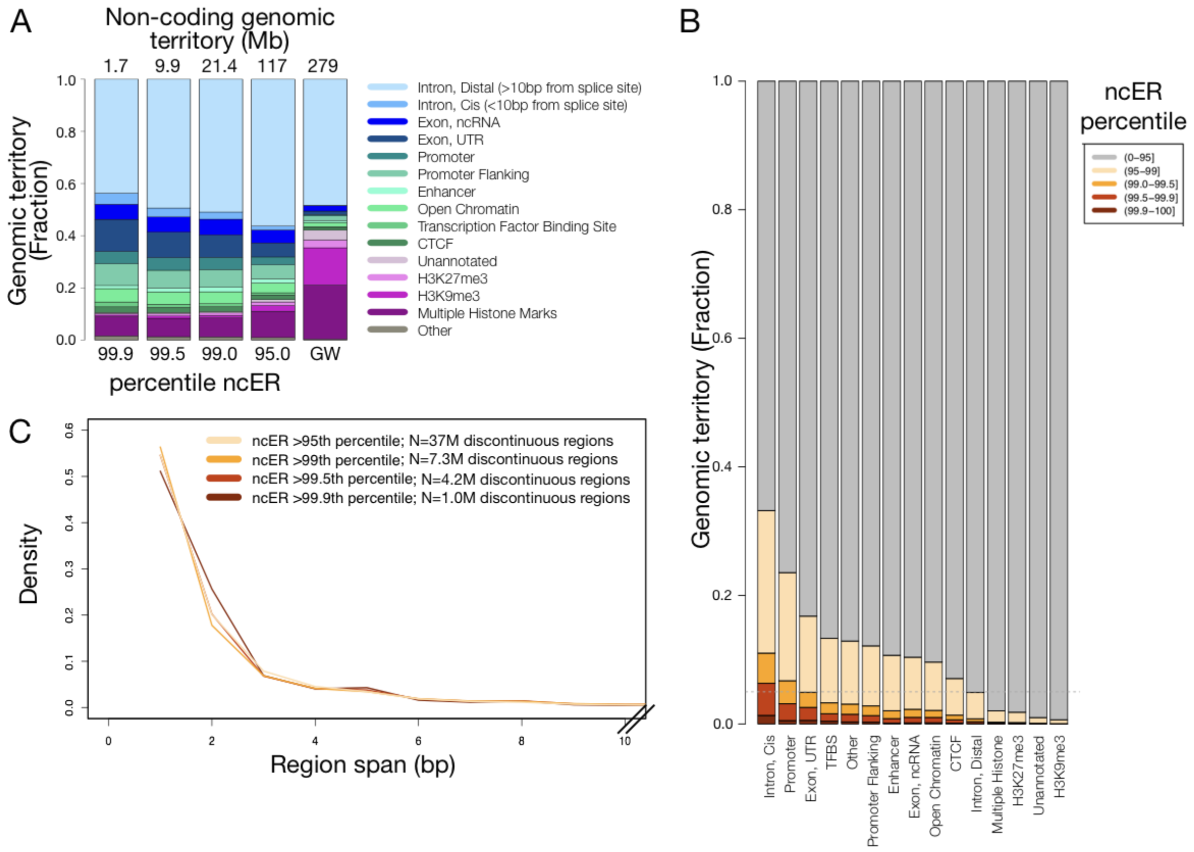

Supplementary Figure 6. The GO terms enrichment for biological processes in high score ncER regions.

The bubble plot represents the significance of the enrichment for a given term (y axis, minus log10(pvalue)) versus the percentage of genes associated with a given biological process that were present in the set of genes with at least one essential promoter bin (x axis). Each circle in the plot represents one biological process. The size of the circles is proportional to the number of genes in the specific GO term class. Only the significant terms are colored. The circles and the rows in the associated table are colored according to the highest-ranking hierarchical term (to facilitate pathway and redundant information detection). When multiple terms could be the highest hierarchical ancestor, the coloring was randomly selected among the multiple possibilities. The associated table provides the name of the top ranked terms. Promoter regions were defined as the 600bp upstream the transcription start site. A modified version of the GOBubble function from the GOplot R package (http://wencke.github.io/) was used to generate this figure.

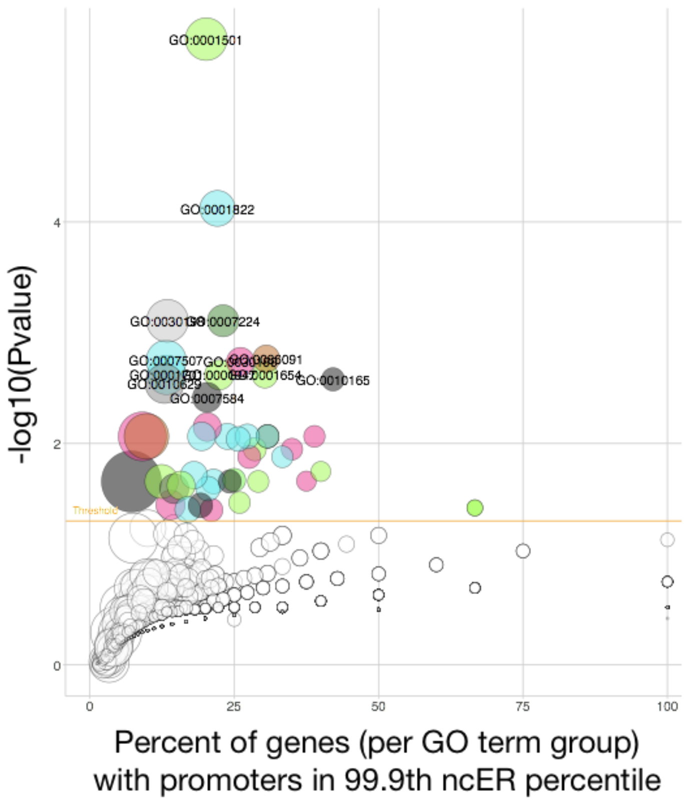

Top enriched terms

GO:0001501	skeletal system development
GO:0001654	eye development
GO:0001701	in utero embryonic development
GO:0001822	kidney development
GO:0001947	heart looping
GO:0007224	smoothened signaling pathway
GO:0007507	heart development
GO:0007584	response to nutrient
GO:0010165	response to X−ray
GO:0010629	negative regulation of gene expression
GO:0030198	extracellular matrix organization
GO:0030199	collagen fibril organization
GO:0086091	regulation of heart rate by cardiac conduction

- multicellular_organismal_process
- developmental_process
- biological_regulation
- cellular_process
- signaling
- response_to_stimulus
- negative_regulation_of_biological_process
- cellular_component_organization_or_biogenesis

Supplementary Figure 7. ncER distribution of GWAS catalog variants.

ncER distribution of (i) GWAS hit SNPs from GWAS catalog (red), keeping the best hit per study per phenotype and a maximum of 1 hit per genomic coordinate, (ii) all common (af>0.05) variants from gnomAD (light grey) and (iii) a random subset of common variants matched to GWAS hits by genomic element distribution and distance to closest splice sites (darkgrey); P values were computed using Kolmogorov–Smirnov test.

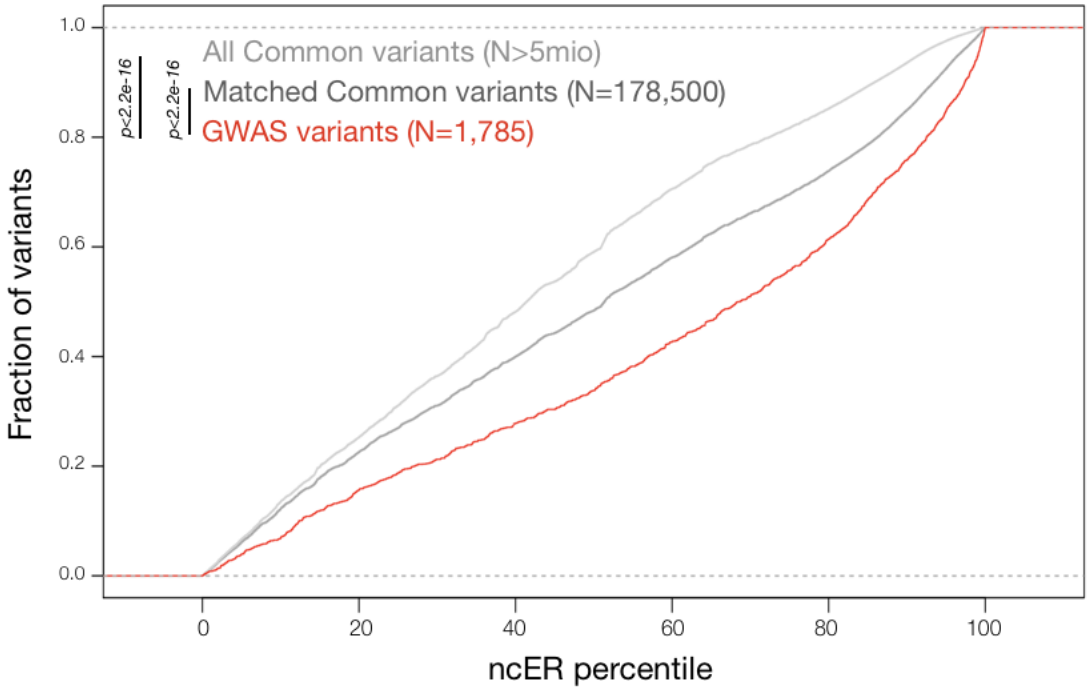

Supplementary Figure 8. Comparison of experimental CRISPRi functional assays with *in silico* predictions of deleteriousness.

Panel A. CRISPRi effect on cell viability (71,404 sgRNA non-coding probe pairs targeting the *MYC* locus) and the corresponding maximum ncER score within the tested region. Accuracy at four ncER thresholds is shown in yellow, orange, red and dark-red respectively for the 95th, 99th, 99.5th and 99.9th ncER percentiles. **Panel B.** Distribution of maximum ncER at different bins of cell viability (0 to lower than -3 log2 fold change). The boxplot's central line represents the median, the bounds represent the 25th and 75th percentile, and the whiskers extend up to 1.5 the interquatile range from the respective bounds. P values were computed with independent 2-group Man-Whitney Unpaired Test. **Panel A.** CRISPRi effect on cell viability (4,177 sgRNA non-coding probe pairs targeting the *GATA1* locus) and the corresponding maximum ncER score within the tested region. Accuracy at four ncER thresholds is shown in yellow, orange, red and dark-red respectively for the 95th, 99th, 99.5th and 99.9th ncER percentiles. **Panel B.** Respective distribution of maximum ncER at different bins of cell viability (0 to lower than -3 log2 fold change). The boxplot's central line represents the median, the bounds represent the 25th and 75th percentile, and the whiskers extend up to 1.5 the interquatile range from the respective bounds. P values were computed with independent 2-group Man-Whitney Unpaired Test. CRISPRi, clustered regularly interspaced short palindromic repeats interference. sgRNA, single guide RNA.

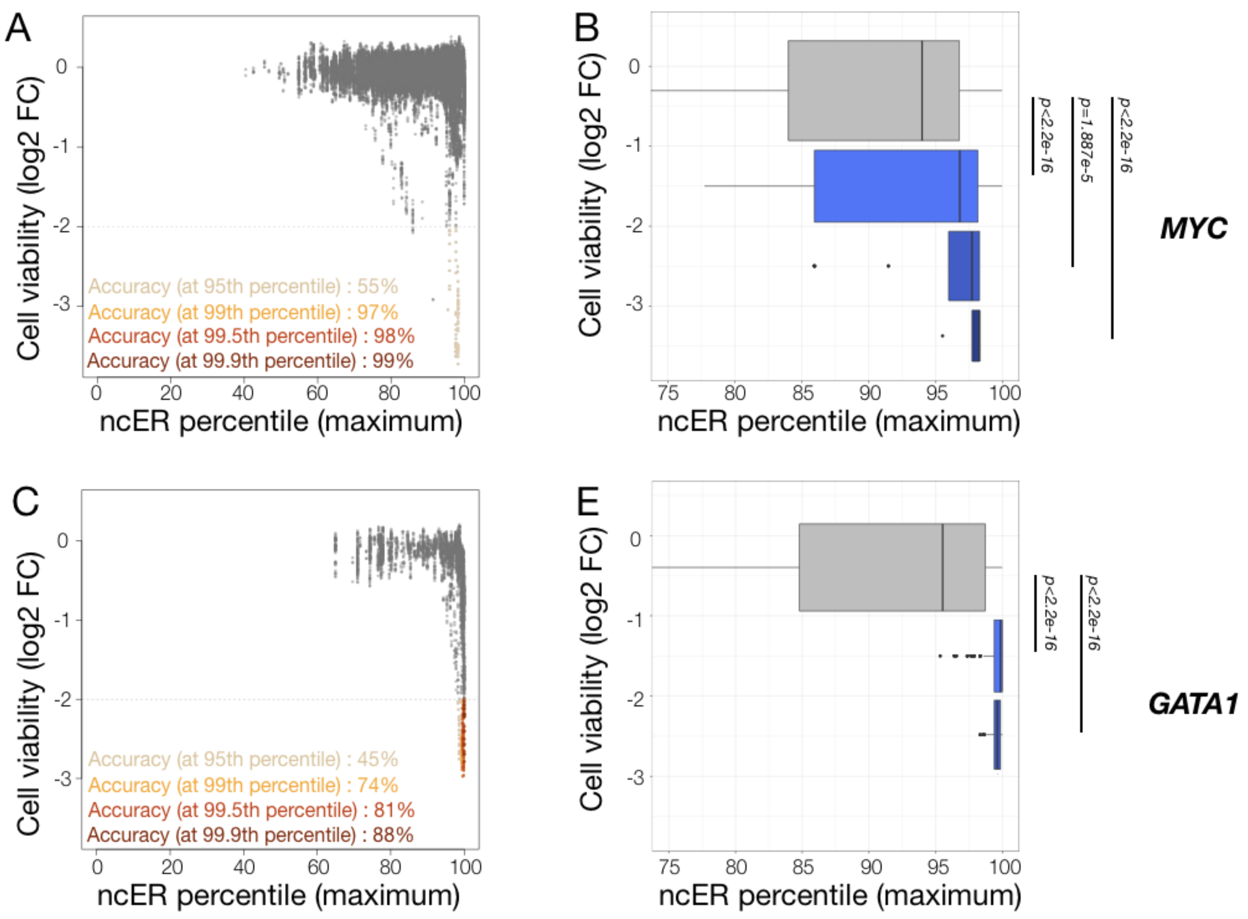
A
B
C
E
Cell viability (log2 FC)
ncER percentile (maximum)
Accuracy (at 95th percentile) : 55%
Accuracy (at 99th percentile) : 97%
Accuracy (at 99.5th percentile) : 98%
Accuracy (at 99.9th percentile) : 99%
p<2.2e-16
p=1.887e-5
p<2.2e-16
MYC
Accuracy (at 95th percentile) : 45%
Accuracy (at 99th percentile) : 74%
Accuracy (at 99.5th percentile) : 81%
Accuracy (at 99.9th percentile) : 88%
p<2.2e-16
p<2.2e-16
GATA1

Supplementary Figure 9. Comparison of experimental CREST-seq functional assays with *in silico* predictions of expression.

Panel A. P values determined by Diao et al. (Nat Methods 14, 629-635 (2017)) comparing *POU5F1* expression in targeted cells versus controls (11,570 sgRNA probe pairs) and the corresponding maximum ncER score within the tested region. Accuracy at four ncER thresholds is shown in yellow, orange, red and dark red respectively for the 95th, 99th, 99.5th and 99.9th ncER percentiles. **Panel B.** Distribution of maximum ncER at different bins of -log10(P value) (up to 5.36 (non-significant), above 5.36 which corresponds to -log10(0.05/11,570)). The boxplot's central line represents the median, the bounds represent the 25th and 75th percentile, and the whiskers extend up to 1.5 the interquatile range from the respective bounds. P values were computed with independent 2-group Mann-Whitney Unpaired Test. sgRNA, single guide RNA.

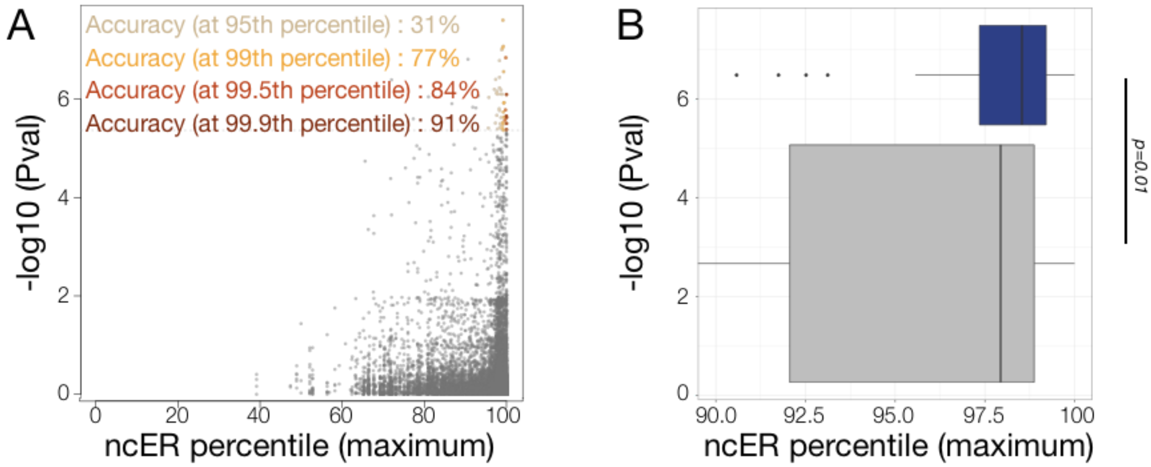

Supplementary Figure 10. CREST-seq peaks enrichment in high score ncER regions.

CREST-seq peaks (N=45, dark blue) display the highest ncER percentile distribution, compared to 100 permutations (grey), each containing 45 regions matched by size to the CREST-seq peaks and from the same genomic locus. The p-value reflects the probability of the median ncER being as high or equal to the one obtained with the functionally confirmed regions (p=1/101). CRESTseq, *cis*-regulatory elements by tiling-deletion and sequencing.

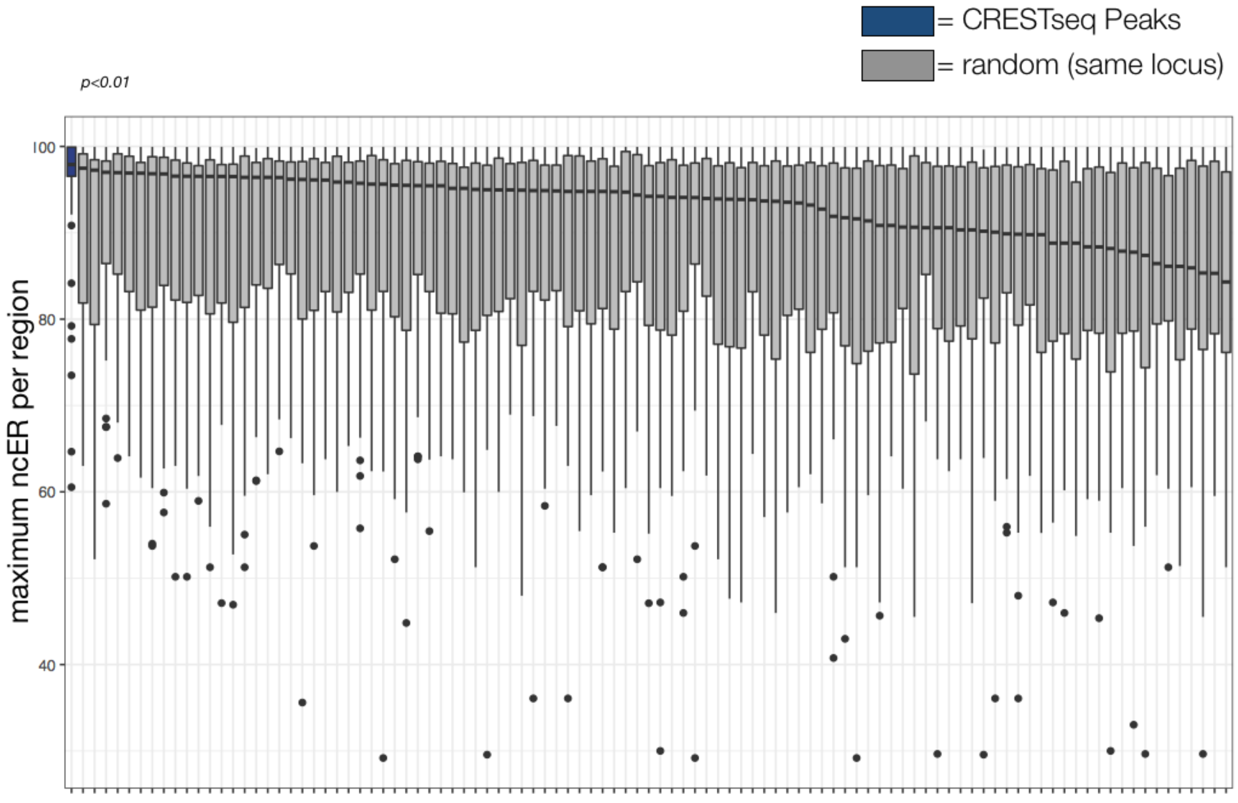

Supplementary Figure 11. Cumulative high score ncER territory within transmitted deletions.

Cumulative distribution function of the fraction of deletions (Y-axis) with a given total number of essential nucleotides (X-axis) per deletion. Essential bins are defined at four different ncER percentile thresholds. Autism and ASD deletions are shown in red (ASD, N=120), control deletions in dark grey (control, N=16) and random size-matched *in silico* deletions extracted genome-wide in light grey (random, N=13,600). The right shift of the red line compared to the grey lines indicates a higher cumulative number of essential nucleotides per deletion in the ASD probands. ASD, Autism Spectrum Disorder.

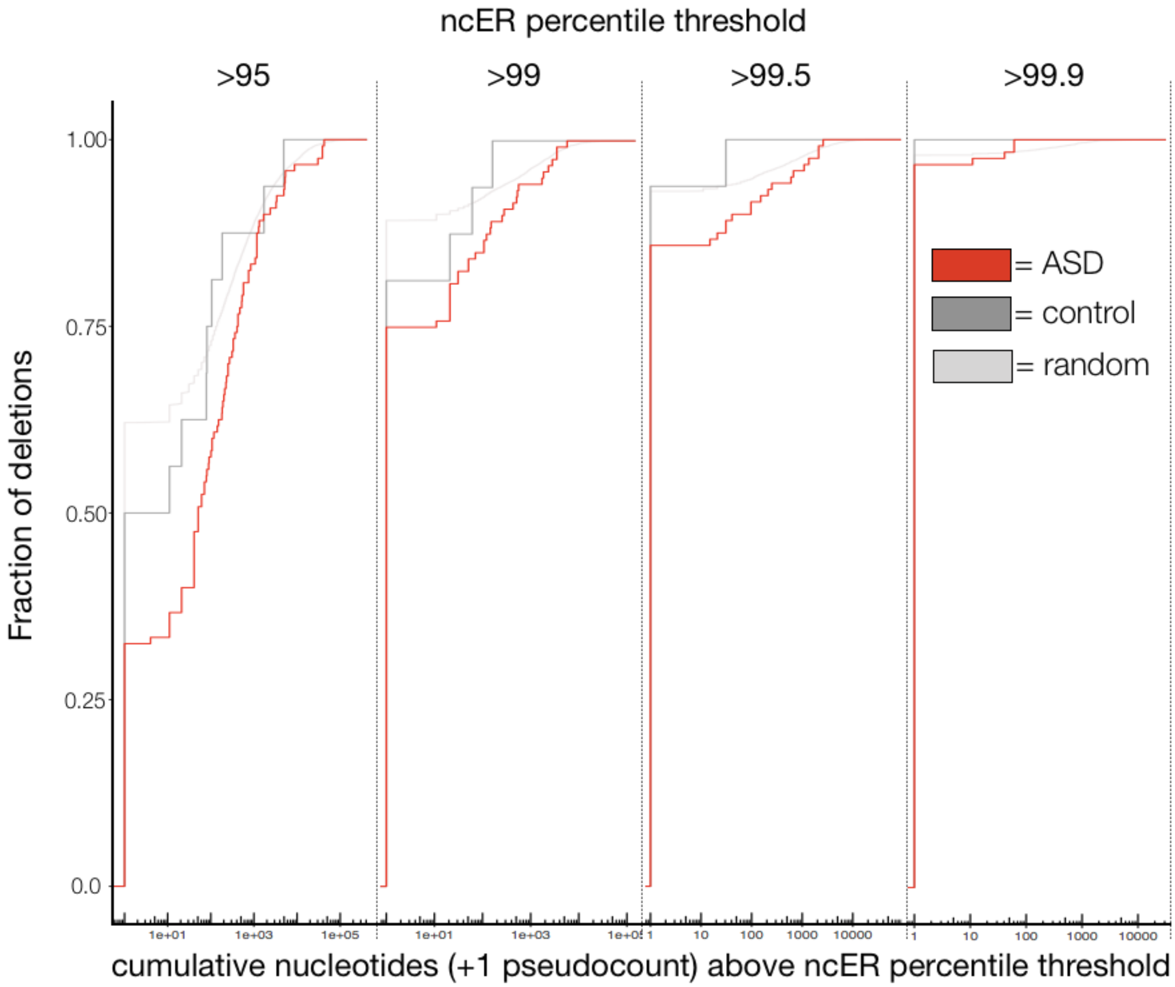

Supplementary Figure 12. Size distribution of Cis-regulatory transmitted deletions.

The transmitted cis-regulatory deletions were split into 4 groups of matched size (with no significant difference in the distribution of control and ASD deletion within the same group size). Deletions present in cases are shown in red, deletions present in controls are shown in grey. The y axis is on logarithmic scale. P values were computed with independent 2-group Man-Whitney Unpaired Test. ASD, Autism Spectrum Disorder

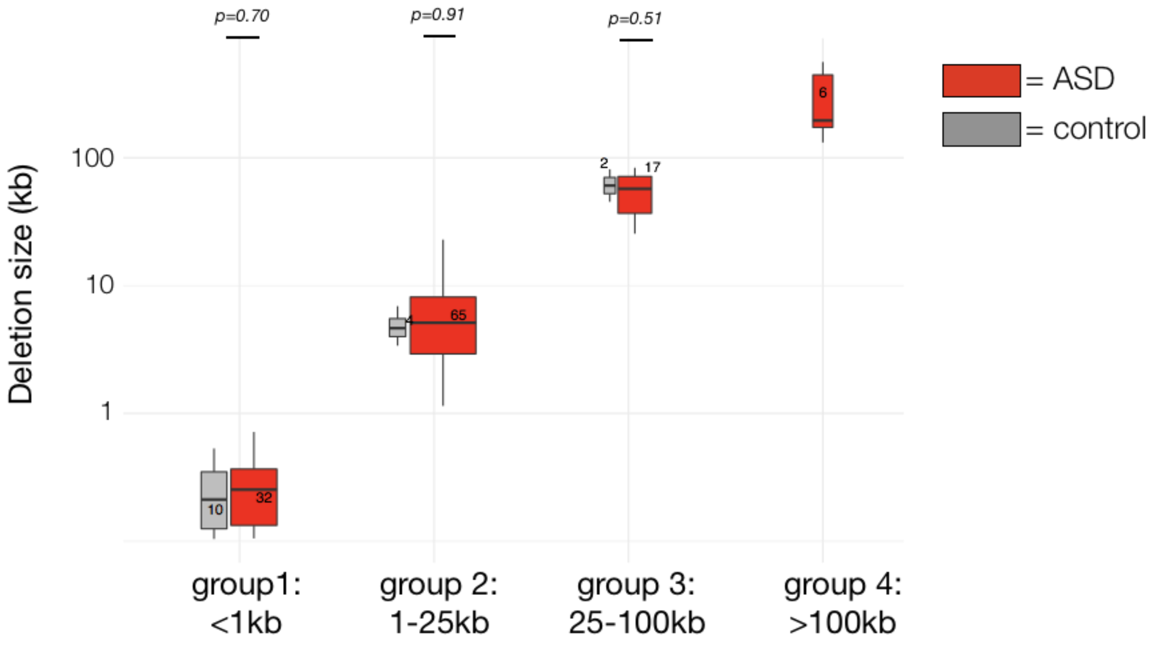

Supplementary Figure 13. Fraction of transmitted deletions with high score ncER functional domains.

Panel A. Fraction <1kb deletions with at least one high score ncER bin. Bins are defined by four different ncER percentile thresholds. Autism and ASD deletions are shown in red ("ASD", N=32), control deletions in dark grey ("control", N=10) and random size-matched *in silico* deletions extracted genome-wide in light grey ("random", N=4,200).
Panel B. Fraction of 1-25kb deletions with at least one high score ncER bin. Bins are defined by four different ncER percentile thresholds. Autism and ASD deletions are shown in red ("ASD", N=65), control deletions in dark grey ("control", N=4) and random size-matched *in silico* deletions extracted genome-wide in light grey ("random", N=6,900). **Panel C.** Fraction of 25-100kb deletions with at least one essential bin. Essential bins are defined by four different ncER percentile thresholds. Autism and ASD deletions are shown in red ("ASD", N=17), control deletions in dark grey ("control", N=2) and random size-matched *in silico* deletions extracted genome-wide in light grey ("random", N=1,900). P values were computed with Fisher Exact Test. ASD, Autism Spectrum Disorder.

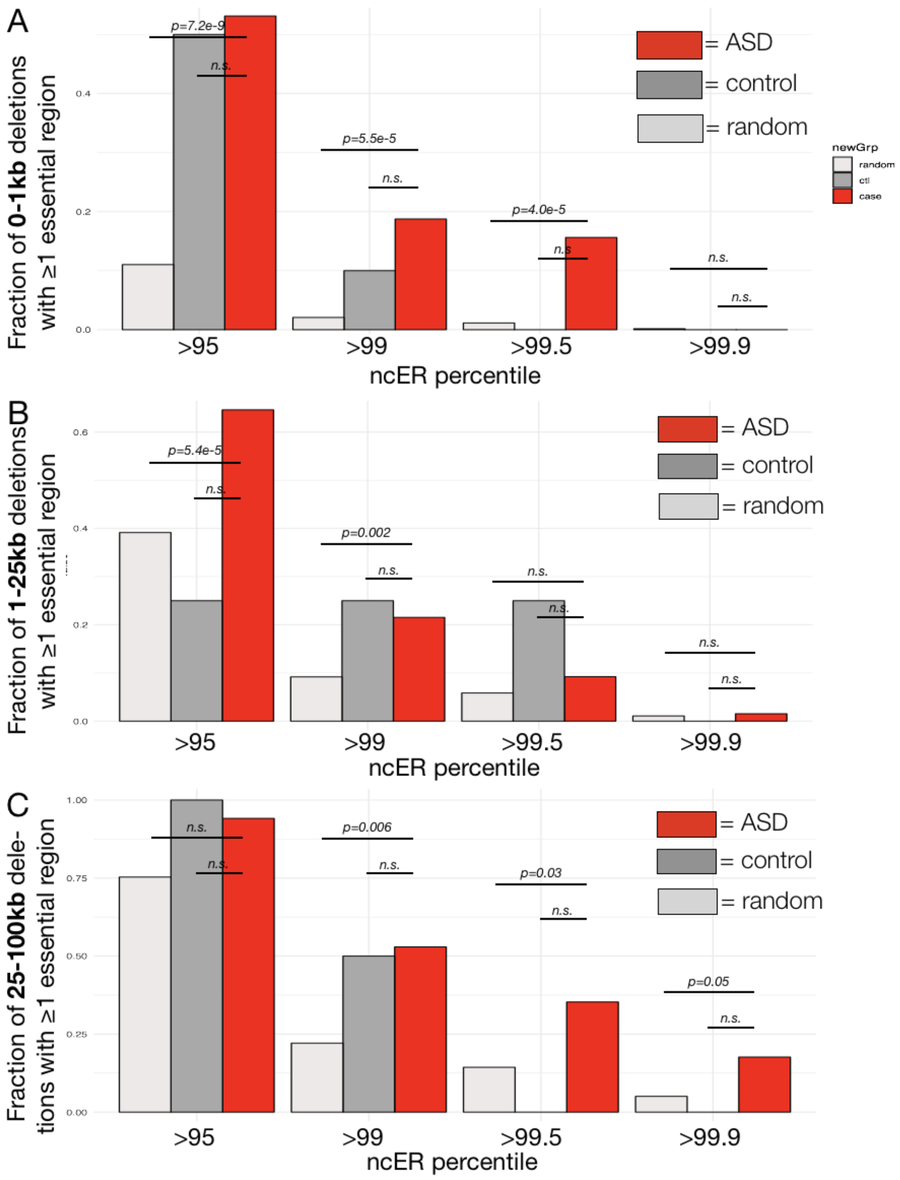
A
B
C
Fraction of 0-1kb deletions with ≥1 essential region
Fraction of 1-25kb deletions with ≥1 essential region
Fraction of 25-100kb deletions with ≥1 essential region
ncER percentile
>95
>99
>99.5
>99.9
= ASD
= control
= random
newGrp
random
ctl
case
p=7.2e-9
p=5.5e-5
p=4.0e-5
p=5.4e-5
p=0.002
p=0.006
p=0.03
p=0.05
n.s.

Supplementary Figure 14. Enrichment in high score ncER regions at mouse functional enhancers.

Functional enhancers (N=9, blue) are placed among the highest ncER percentile distribution compared to 100 permutations (grey), each containing 9 regions matched by size to the enhancers and issued from the same genomic locus. The p-value reflects the probability of the median ncER being as high or equal to the one obtained with the functionally confirmed regions (p=4/101).

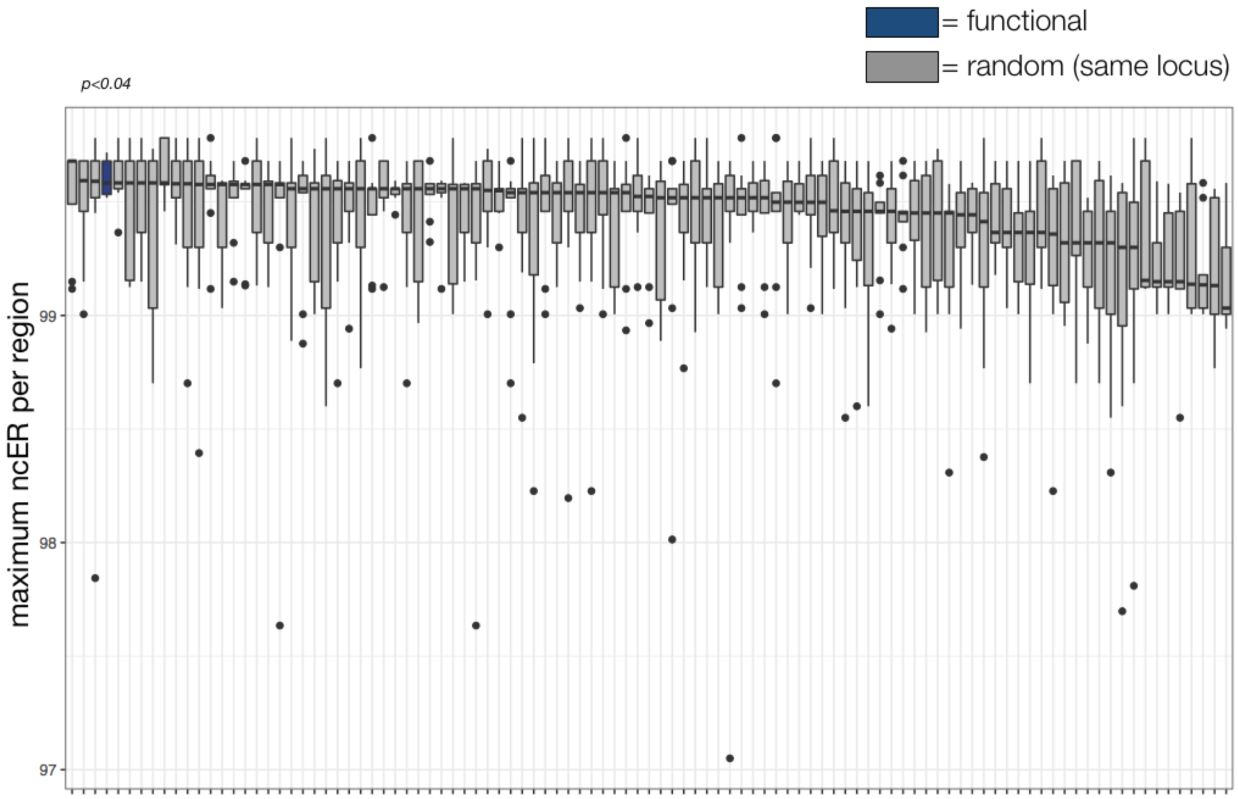

Supplementary Figure 15. ncER input feature signals across the IHH locus.

Every input feature score was normalized between 0 (least likely essential/functional in blue) and 1 (most likely essential/functional). White areas indicate no values at those positions. Input features that do not include the locus are not shown. Input features are displayed in the same order than in **Figure 1C**. Upper panel pictogram is adapted from[37]. Dark red ovale shapes represent putative enhancers.

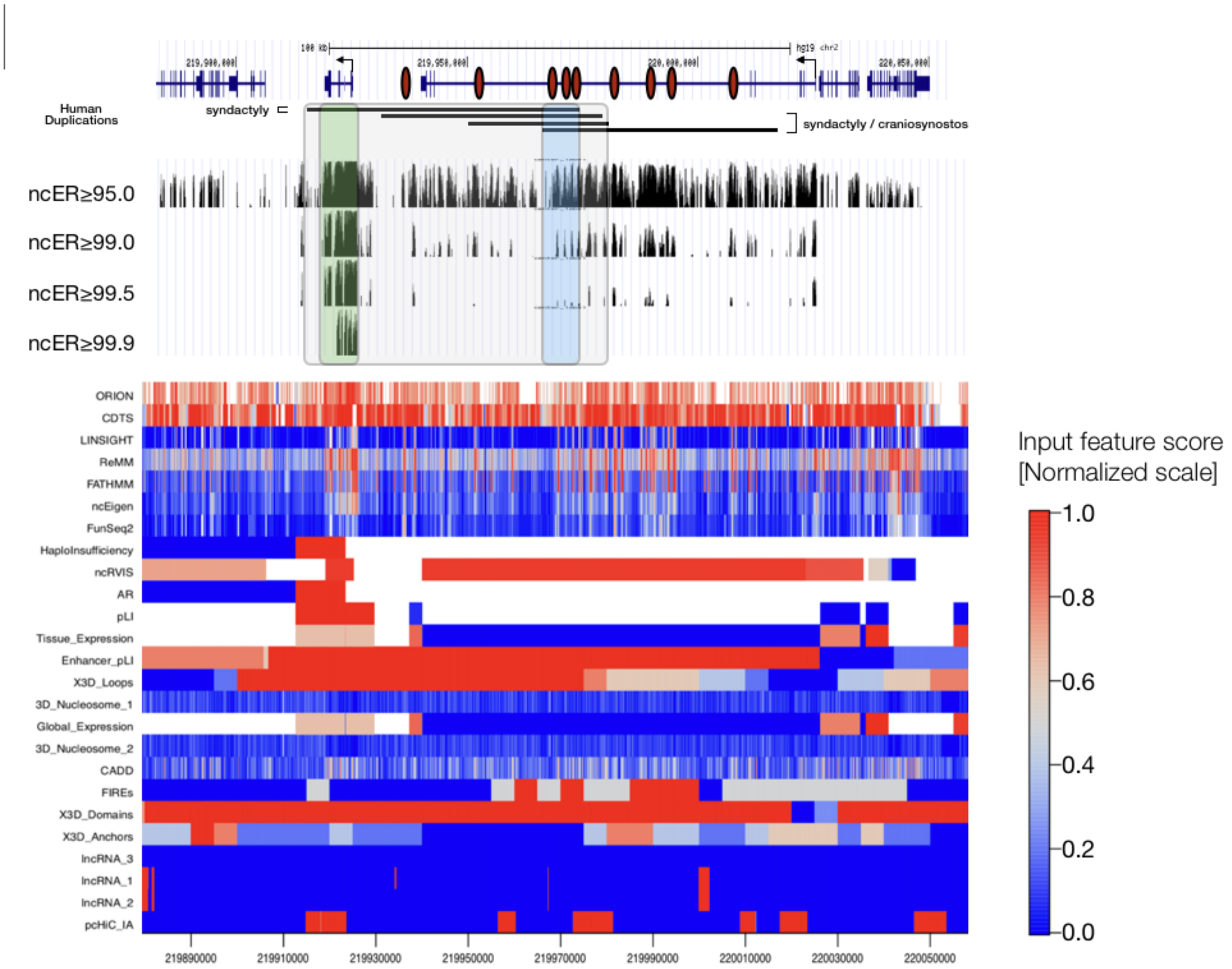

Supplementary Table 1. Predictive performance and accuracy of ncER compared to CRISPRi functional assays.

condition	ncER percentile threshold	sensitivity	specificity	PPV	NPV	accuracy
cell viability (log2 FC) <= -3	99.9	0	98.5	0	99.95	98.44
cell viability (log2 FC) <= -3	99.5	0	97.05	0	99.95	97
cell viability (log2 FC) <= -3	99	0	95.9	0	99.95	95.85
cell viability (log2 FC) <= -3	95	100	54.32	0.11	100	54.34
cell viability (log2 FC) <= -2	99.9	10.59	98.53	2.37	99.69	98.23
cell viability (log2 FC) <= -2	99.5	45.88	97.19	5.24	99.81	97.02
cell viability (log2 FC) <= -2	99	62.35	96.1	5.13	99.87	95.99
cell viability (log2 FC) <= -2	95	97.25	54.46	0.72	99.98	54.61
cell viability (log2 FC) <= -1	99.9	21.51	98.77	19	98.94	97.74
cell viability (log2 FC) <= -1	99.5	42.13	97.57	18.94	99.21	96.84
cell viability (log2 FC) <= -1	99	53.09	96.56	17.2	99.35	95.99
cell viability (log2 FC) <= -1	95	89.44	54.88	2.6	99.74	55.34

Supplementary Table 2. **Predictive performance and accuracy of ncER compared to CREST-seq functional assays.** The p-value cutoff of 5.36 corresponds to the Bonferroni adjusted p value in the original study.

condition	ncER percentile threshold	sensitivity	specificity	PPV	NPV	accuracy
-log10(pval)>=5.36	99.9	9.43	90.93	0.48	99.54	90.55
-log10(pval)>=5.36	99.5	15.09	84.08	0.43	99.54	83.77
-log10(pval)>=5.36	99	37.74	76.85	0.74	99.63	76.67
-log10(pval)>=5.36	95	83.02	30.80	0.55	99.75	31.04
