# Supplementary material for: Ranking of non-coding pathogenic variants and putative essential regions of the human genome
Source: Nat Commun. 2019 Nov 20;10:5241. doi: 10.1038/s41467-019-13212-3 (PMC6868241; doi:10.1038/s41467-019-13212-3)
Supplement: Supplementary file 6 — Reporting Summary [file 41467_2019_13212_MOESM6_ESM.pdf]

nature research

Corresponding author(s): Amalio Telenti and Julia di Iulio

Last updated by author(s): Oct 23, 2019

Reporting Summary

Nature Research wishes to improve the reproducibility of the work that we publish. This form provides structure for consistency and transparency in reporting. For further information on Nature Research policies, see Authors & Referees and the Editorial Policy Checklist .

Statistics

For all statistical analyses, confirm that the following items are present in the figure legend, table legend, main text, or Methods section.

n/a	Confirmed	
[ ]	[x]	The exact sample size (*n*) for each experimental group/condition, given as a discrete number and unit of measurement
[x]	[ ]	A statement on whether measurements were taken from distinct samples or whether the same sample was measured repeatedly
[ ]	[x]	The statistical test(s) used AND whether they are one- or two-sided<br>*Only common tests should be described solely by name; describe more complex techniques in the Methods section.*
[ ]	[x]	A description of all covariates tested
[x]	[ ]	A description of any assumptions or corrections, such as tests of normality and adjustment for multiple comparisons
[x]	[ ]	A full description of the statistical parameters including central tendency (e.g. means) or other basic estimates (e.g. regression coefficient) AND variation (e.g. standard deviation) or associated estimates of uncertainty (e.g. confidence intervals)
[x]	[ ]	For null hypothesis testing, the test statistic (e.g. *F*, *t*, *r*) with confidence intervals, effect sizes, degrees of freedom and *P* value noted<br>*Give P values as exact values whenever suitable.*
[x]	[ ]	For Bayesian analysis, information on the choice of priors and Markov chain Monte Carlo settings
[x]	[ ]	For hierarchical and complex designs, identification of the appropriate level for tests and full reporting of outcomes
[x]	[ ]	Estimates of effect sizes (e.g. Cohen's *d*, Pearson's *r*), indicating how they were calculated

Our web collection on statistics for biologists contains articles on many of the points above.

Software and code

Policy information about availability of computer code

Data collection	The data collection is described in "External Datasets" section and Supplementary Table 1.
Data analysis	Code for the model is provided in https://github.com/TelentiLab/ncER_datasets. The figures were plotted with R v3.3.2 (https://www.R-project.org/) and Python (v.2.7.13). Data mining was performed using unix command line.

For manuscripts utilizing custom algorithms or software that are central to the research but not yet described in published literature, software must be made available to editors/reviewers. We strongly encourage code deposition in a community repository (e.g. GitHub). See the Nature Research guidelines for submitting code & software for further information.

Data

Policy information about availability of data

All manuscripts must include a data availability statement. This statement should provide the following information, where applicable:

- Accession codes, unique identifiers, or web links for publicly available datasets
- A list of figures that have associated raw data
- A description of any restrictions on data availability

Genome-wide ncER scores are also provided for download at https://github.com/TelentiLab/ncER_datasets and can be browsed directly at OMNI (https://www.ai-omni.com/).

Field-specific reporting

Please select the one below that is the best fit for your research. If you are not sure, read the appropriate sections before making your selection.

Life sciences study design

All studies must disclose on these points even when the disclosure is negative.

Sample size	No sample size calculation was done. To our knowledge, we used all non-coding pathogenic variants available at the time of analysis.
Data exclusions	we excluded non-coding pathogenic variants that were within 10bp of any splice site.
Replication	we validated the model on 2 generalization sets of non-coding pathogenic variants (N=77 and N=209 respectively) as well as on a set of Mendelian trait associated non-coding pathogenic variants (N=137)
Randomization	the model is trained and tested on non-overlapping chromosomic regions to counteract the potential over-training of some genes. Chromosomic regions used for training were randomly selected to reach 80% of the data and the remaining 20% was used for testing. Control variants were then matched to pathogenic variants according to the genomic element distribution and distance to splice site.
Blinding	Not relevant

Reporting for specific materials, systems and methods

We require information from authors about some types of materials, experimental systems and methods used in many studies. Here, indicate whether each material, system or method listed is relevant to your study. If you are not sure if a list item applies to your research, read the appropriate section before selecting a response.

Materials & experimental systems

n/a	Involved in the study
☒	☐ Antibodies
☒	☐ Eukaryotic cell lines
☒	☐ Palaeontology
☒	☐ Animals and other organisms
☒	☐ Human research participants
☒	☐ Clinical data

Methods

n/a	Involved in the study
☒	☐ ChIP-seq
☒	☐ Flow cytometry
☒	☐ MRI-based neuroimaging
