# Supplementary material for: Ranking of non-coding pathogenic variants and putative essential regions of the human genome
Source: Nat Commun. 2019 Nov 20;10:5241. doi: 10.1038/s41467-019-13212-3 (PMC6868241; doi:10.1038/s41467-019-13212-3)
Supplement: Supplementary file 7 — Description of Additional Supplementary Files [file 41467_2019_13212_MOESM7_ESM.pdf]

Title: Supplementary Data 1.
Description: Pathogenic variants' genomic coordinates. Provided as separate file.

Title: Supplementary Data 2.
Description: Input feature description and accession links. Provided as separate file.

Title: Supplementary Data 3.
Description: Mendelian variants, disease and heritability model. Provided as separate file
